# Computational study of conformational interconversion of an amyloid β double layer system

**DOI:** 10.1039/d5ra08004e

**Published:** 2025-11-03

**Authors:** Yasuhiro Oishi, Motoharu Kitatani, Kichitaro Nakajima, Hirotsugu Ogi, Koichi Kusakabe

**Affiliations:** a Graduate School of Science, University of Hyogo 3-2-1 Koto, Kamigori-cho, Ako-gun 678-1297 Japan rk23m002@guh.u-hyogo.ac.jp; b Graduate School of Engineering, The University of Osaka 2-1 Yamadaoka Suita Osaka 565-0871 Japan

## Abstract

The formation of amyloid fibrils comprising amyloid β (Aβ) peptides is associated with the pathology of Alzheimer's disease. In this study, we theoretically investigated conformational changes of a flat double-layer structure of two Aβ^20−34^ peptides using the density functional theory calculation. Several twisted conformations were identified as local energy minima in which a part of the peptide chain bends upward while the rest remains bound to the lower Aβ^20−34^ monomer. Flat-to-twisted conformational transition exhibited endothermic behavior, with endothermic energy increasing as more backbone hydrogen bonds were broken. In addition, the loss of van der Waals interaction from the hydrophobic sidechain contributed to endothermicity. The nudged elastic band method was applied to analyze the potential energy surface connecting the flat and twisted conformations. Comparison of the activation barriers between different twisted conformations revealed that certain twisted conformations returned relatively easily to the flat conformation, whereas others encountered a higher activation barrier and reverted less readily. Detailed structural analysis revealed that the twisted conformation's propensity to return originates from the local steric hindrance imposed by the sidechain near the torsional axis.

## Abstract 2

The formation of amyloid fibrils comprising amyloid β (Aβ) peptides is associated with the pathology of Alzheimer's disease.

## Graphical abstract

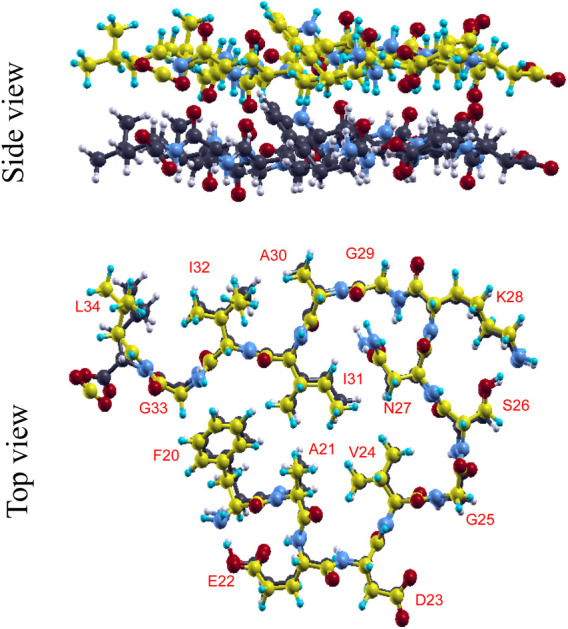

## 1 Introduction

Protein aggregation often results in the formation of amyloid fibrils, which exhibit a characteristic needle-like morphology^1^ and play a crucial role in the amyloidosis pathology.^2^ In particular, Alzheimer's disease is associated with amyloid fibrils comprising amyloid β (Aβ) peptides.^3,4^ The Aβ peptide is generated through the cleavage of the amyloid precursor protein by β- and γ-secretases,^5,6^ and the generated peptide exhibits a tendency to self-assemble, finally forming fibrillar structures.

Amyloid fibril structures have been extensively characterized by experimental techniques, such as X-ray diffraction,^7,8^ solid-state nuclear magnetic resonance,^9–14^ and Cryogenic Electron Microscopy.^15–19^ Theoretically, the amyloid-like peptide molecules have been extensively investigated using the density functional theory (DFT).^20–23^ These studies focused on an accurate description of the hydrogen bond (HB) interaction. Several works have examined the role of HBs in amyloid fibril stability.^20–22^ Indeed, the energetics have been discussed based on the association energy of peptides in crystalline forms.^20,21^ In contrast, conformational changes of an individual peptide molecule in amyloid fibrils remain an area requiring further theoretical investigation.

Generally, conformational changes in protein molecules involve cooperative molecular motions. In this study, we consider the torsional motion around the backbone dihedral angles that are the Ramachandran dihedral angles of *ψ* and *ϕ* defined for the C–C and N–C single bonds in the peptide unit. Actually, the potential energy surface (PES) associated with these torsions has been explored in other peptide systems.^24–27^ We specifically treat possible torsional conformational changes within computer experiments. The energetics of the torsional motion is explored using the nudged elastic band (NEB) method based on DFT. The torsional motions in this study are particularly relevant for our discussion.

We considered a peptide encoding 20–34 residues of the Aβ peptide (Aβ^20−34^) as a monomer (PDB ID: 6OIZ). This peptide is known to form a crystallized structure in a parallel β-sheet conformation observed by the electron diffraction experiment.^28^ This peptide adopts a β-helix-like turn, which is a common structural motif observed in the full-length Aβ (Aβ^1−42^).^28^ We treat a double-layer structure in which two Aβ^20−34^ monomers are aligned in a parallel β sheet. As discussed in the following sections, the study of this short-length peptide can provide us with information on conformational changes in an amyloid fibril.

The stability and structural features resulting from torsion are examined in detail. We reveal the role of local steric hindrance in creating an activation barrier associated with torsional motion. Based on these findings, we discuss possible single-molecule conformational changes in an amyloid fibril.

## 2 Computational methods

### 2.1 Investigated conformations of a double-layer structure

The direction of torsion and the possible torsional axis are limited due to intermolecular and intramolecular steric hindrances. Some torsions result in a structure in which a part of the upper monomer's peptide chain separates from the lower monomer, while the remaining segment remains stacked through a parallel β-sheet. We define such a structure as a twisted conformation. In contrast, we define a structure in which the upper monomer is fully stacked on the lower monomer *via* a parallel β-sheet as a flat conformation.

### 2.2 Potential energy surface calculation

In our calculation based on DFT, we employed the PWscf code of Quantum ESPRESSO.^29–31^ For the exchange–correlation functional, the PBEsol functional^32^ together with the DFT-D3 correlation^33^ was used to describe the van der Waals interaction. Ultrasoft pseudopotentials^34^ with an energy cutoff of 25(250) Ry for wavefunction (charge density) expansion were employed. Structural optimization was performed for the flat and twisted conformation until the Hellman-Feynman force acting on each atom was less than 10^−5^ Ry per Bohr. The NEB method^35^ was employed to search for the pathway connecting the flat and twisted conformation. The calculation was performed only at the gamma-point for an isolated dimer system in a cell with a size of 45 × 45 × 40 Å^3^. Validation of the cell size was confirmed by performing a structural optimization for a representative twisted conformation—the structure exhibiting the largest separation of the upper monomer peptide chain—in the expanded cell size of 50 × 50 × 50 Å^3^. The atomic structure showed no substantial changes compared to that with the original cell, and the difference in the relative energy of the twisted to the flat conformation was only 0.008 eV. These results indicate that the original cell size (45 × 45 × 40 Å^3^) is sufficient to represent the isolated double-layer system without significant spurious interactions. We used Xcrysden^36^ to measure the interatomic distance and display the atomic structure.

## 3 Results

This section presents the structural and energetic analysis of the Aβ^20−34^ dimer. The structure of the flat conformation is first described, after which the twisted conformations and their relative energies to the flat conformation are presented. The energy trend among the different twisted conformations is then explained based on their structural differences. In addition, the PESs connecting the flat and twisted conformations obtained using the NEB calculation are presented. Activation barriers associated with each torsion are compared, and the origins of the barrier heights are analyzed.

### 3.1 Structure of the flat conformation

**Fig. 1:**
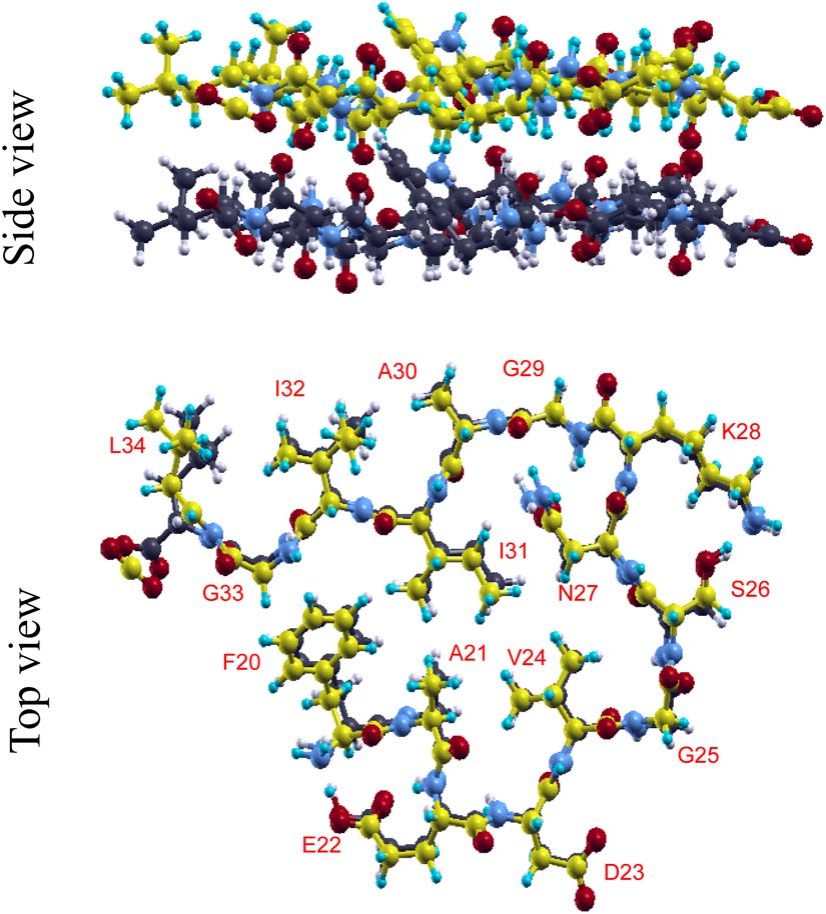
Side and top views of the optimized structure of the flat conformation. Throughout this paper, the carbon and hydrogen atoms in the upper monomer are shown as yellow and cyan spheres, respectively, while the carbon and hydrogen atoms in the lower monomer are shown as black and white spheres, respectively. The nitrogen and oxygen atoms are shown as blue and red spheres, respectively. The amino acid label was added as red letters near the corresponding sidechains. Amino acid residues are denoted using the standard single-letter code with their position in the Aβ(1–42) sequence (*e.g.*, F20 refers to phenylalanine at position 20) throughout this paper.

Fig. 1 shows the optimized structure of the flat conformation. The interlayer distance between the upper and lower monomers was 4.817 Å, which is close to the experimental value of 4.78 Å^28^. The two monomers are tightly connected through 13 HBs, of which 12HBs involve mainchain atoms and one HB involves the sidechains of N27 residue. As an intermolecular HB, we adopted the criteria for HB employed in the literature;^37^ a HB was considered present if the distance between the donor atom (D) and acceptor atom (A) was less than or equal to 3.6 Å, and the D–H ⋯ A angle was greater than or equal to 120°.

The Aβ^20−34^ peptide exhibits a nearly periodic meandering peptide structure; bends constituting the meandering are formed in every segment containing three consecutive bonds (C–C, CO–NH, and N–C bonds). A segment containing the boundary between the *i*-th and *i* + 1-th amino acid residues is called an *i – i* + 1 segment.

### 3.2 Structure and stability of the twisted conformation

**Fig. 2:**
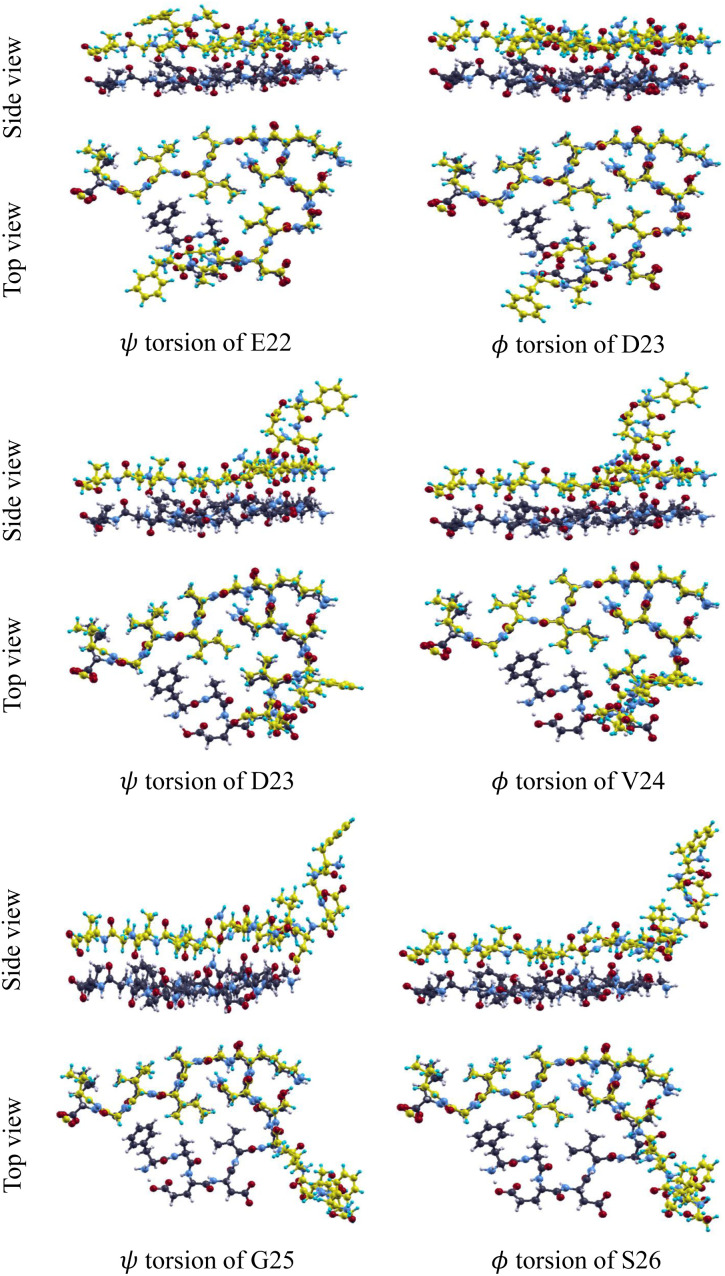
Side and top views of the optimized structure of the twisted conformation for each torsion.

Fig. 2 shows the optimized structures of the twisted conformation. Each twisted conformation is labeled according to the axis around which the structure is twisted using the residue and Ramachandran angle. For example, “*ψ* torsion of E22” refers to torsion around the C–C bond at the E22 residue.

**Fig. 3:**
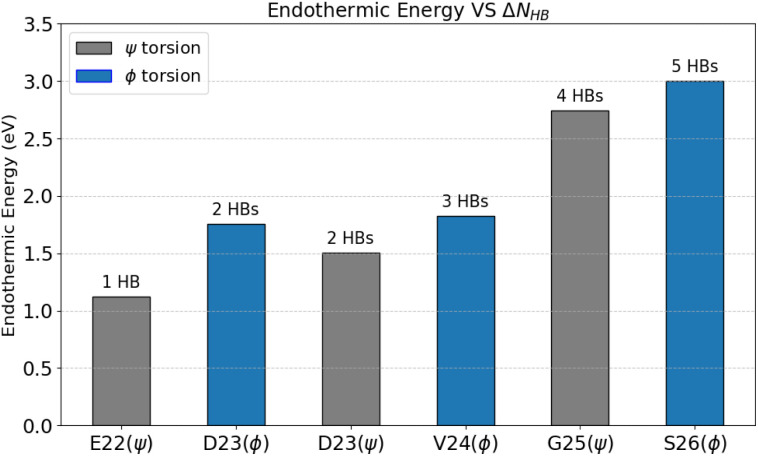
Energy of the twisted conformation relative to the flat conformation for each torsion. The amino acid residue shown on the horizontal axis corresponds to the residue containing the torsional axis. The gray and blue bars represent the energies resulting from *ψ* and *ϕ* torsions, respectively. On top of each bar graph, the number of mainchain hydrogen bonds broken due to each torsion (Δ*N*_HB_) is indicated.

Fig. 3 summarizes the energies of the twisted conformations relative to the flat conformation, showing that all twisted conformations are less stable than the flat conformation. The energy of the twisted conformations increased with the length of the peptide chain lifted under torsion, except for the *ϕ* torsion of D23.

For each twisted conformation, all intermolecular mainchain HBs located from the torsional axis to the N-terminus in the flat conformation were broken. The number of broken HBs is denoted as Δ*N*_HB_ and is shown at the top of each bar graph in Fig. 3. A roughly proportional relationship between endothermic energy and Δ*N*_HB_ was observed, except for the *ϕ* torsion of D23.

Here, we focus on the energy difference between *ψ* and *ϕ* torsions within the same segment (*ψ* torsion of E22 *vs. ϕ* torsion of D23, *ψ* torsion of D23 *vs. ϕ* torsion of V24, and *ψ* torsion of G25 *vs. ϕ* torsion of S26). For each segment, the endothermic energy of the twisted conformation caused by *ϕ* torsion exceeded that caused by *ψ* torsion. The common reason for this is that the Δ*N*_HB_ is one more in *ϕ* torsion than in *ψ* torsion for each segment. The energy difference between *ϕ* and *ψ* torsion was 0.64, 0.32, and 0.26 eV for the 22–23, 23–24, and 25–26 segments, respectively.

The largest energy difference in the 22–23 segment (0.64 eV) arises from repulsion between the mainchain carbonyl oxygen atoms of the upper and lower monomers in the 22–23 segment. Unlike the *ψ* torsion, for the *ϕ* torsion, the oxygen atom in the 22–23 segment of the upper monomer approaches the oxygen atom in the same segment of the lower layer. In the *ϕ* torsion, the distance between the oxygen atoms is 4.7783 Å in the flat conformation, whereas in the twisted conformation, it is reduced to 2.9932 Å. This proximity seems to destabilize the twisted conformation caused by the *ϕ* torsion of D23.

Another type of interaction, such as the van der Waals interaction, also affects endothermic energy. This notably reflects two energy trends: the endothermic energy of the *ψ* torsion of E22 and the energy difference between the *ϕ* torsion of V24 and the *ψ* torsion of G25. First, we discuss the *ψ* torsion of E22. The endothermic energy is 1.12 eV despite only one HB being broken in this torsion. The relationship between endothermic energy and Δ*N*_HB_ indicates that the torsion-induced energy changes cannot be explained solely by Δ*N*_HB_. All calculated torsions involve the breakage of π-stacking of the F20 sidechain, stabilizing the flat conformation *via* the van der Waals interaction. This breakage partly explains why the twisted conformation arising from the *ψ* torsion of E22 has such a high energy, even though only one HB is broken. Second, the energy difference between the *ϕ* torsion of V24 and the *ψ* torsion of G25 reaches 0.92 eV, even though the corresponding difference in the Δ*N*_HB_ is one. A possible explanation to this is the van der Waals interaction arising from the sidechain of V24. In the flat conformation, the V24 sidechain of the upper monomer faces the sidechains of hydrophobic residues of the upper monomer, such as I31, as well as the V24 sidechain of the lower monomer. These contacts may enhance the stability of the flat conformation through van der Waals interactions. In the *ψ* torsion of G25, the V24 sidechain in the upper monomer is separated from these sidechains, unlike in the *ϕ* torsion of V24. Thus, the loss of the van der Waals interaction that stabilizes the flat conformation is greater in the *ψ* torsion of G25 than in the *ϕ* torsion of V24, which seems to increase the energy difference.

Overall, the intermolecular HB and the van der Waals interaction from the hydrophobic sidechain stabilized the flat conformation. The results thus far indicate that when a torsional motion disrupts a greater number of such interactions, the resulting twisted conformation becomes less stable, thereby increasing endothermic energy. Additionally, in torsions where the carbonyl oxygen atoms in the upper and lower monomers approach, the twisted conformation becomes further destabilized. These factors make the transition from the flat to the twisted conformation less probable. Although our study focuses on torsions that lead to the lifting of the peptide chain from the N-terminus, similar results are expected for torsions that lift the peptide chain from the C-terminus.

### 3.3 Pathway and activation barrier for torsional motion

**Fig. 4:**
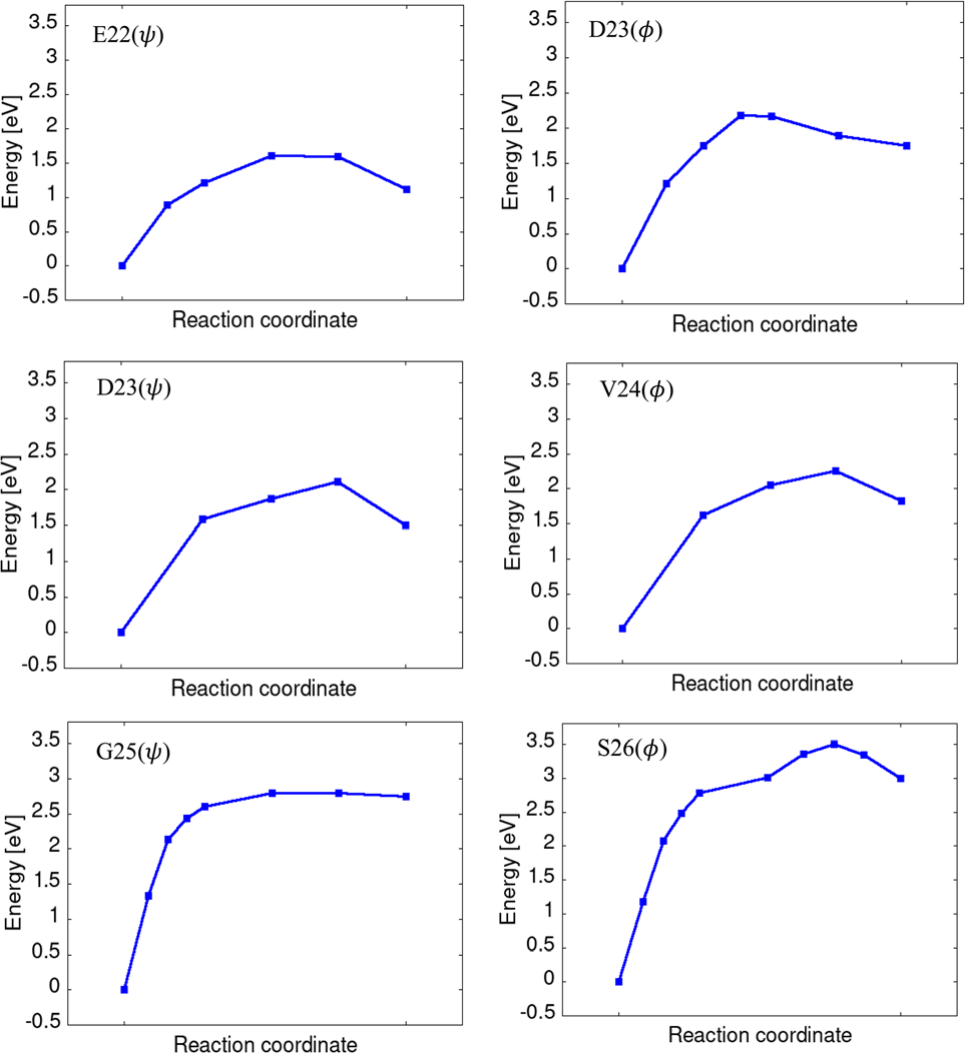
Calculated potential energy surface for each torsion. The energy of the flat conformation was set as a reference (zero energy).

Here, we discuss the reaction pathway connecting the flat and twisted conformations obtained using the NEB calculation. Fig. 4 shows the PES connecting the flat and twisted conformations, with the energy of the flat conformation taken as the reference. All six torsions exhibited endothermic behavior. First, we focus on the difference in the curve of PES between *ψ* and *ϕ* torsions within the same segment. For the 22–23 segment, the energy increase was larger for the *ϕ* torsion of D23 than for the *ψ* torsion of E22, arising from the proximity between the oxygen atoms, as discussed previously. For the 23–24 segment, the *ψ* torsion of D23 and *ϕ* torsion of V24 showed a similar PES curve. The slightly larger energy increase in the *ϕ* torsion of V24 than in the *ψ* torsion of D23 arises from the difference in the Δ*N*_HB_.

The PESs for the *ψ* torsion of G25 and the *ϕ* torsion of S26 showed a similar energy curve in the early part of the pathway. However, in the latter part, the PESs showed different energy curves; in the *ψ* torsion of G25, the curve became relatively flat, while in the *ϕ* torsion of S26, the energy continued to increase. We observed that in the early part of the pathway, both torsions involved the breakage of the four mainchain HBs. In the latter pathway of the *ϕ* torsion of S26, unlike the *ψ* torsion of G25, additional breakage of HB occurs in the 25–26 segment. This additional HB breakage likely contributes to the further increase in energy observed in the PES for the *ϕ* torsion of S26.

Next, we discuss the activation barrier that appears on the PES. The activation barrier for twisted-to-flat conformational transition represents the propensity of the former to revert to the latter. For each PES, an activation barrier for the twisted-to-flat conformational transition was confirmed and summarized in Table 1. The height of these barriers ranged from approximately 0.4 to 0.6 eV, except for the *ψ* torsion of G25, which exhibited a significantly lower barrier of only 0.05 eV.

**Table 1:** The activation barriers from the twisted to the flat conformation for each torsion are summarized. We also present the relevant distance between each atom pair in the upper and lower monomers, which is considered to be related to intermolecular steric hindrance, at both the activation barrier top and the local minimum for each torsional pathway. For the *ψ* torsion of E22 and the *ϕ* torsion of D23, the distances between the hydrogen atom contained in the E22 sidechain and the hydrogen atom in the lower monomer are shown. For the *ψ* torsion of D23 and the *ϕ* torsion of D24, the distances between the oxygen atoms of the carboxyl group in the D23 sidechain and the mainchain carbonyl oxygen atom of the 22–23 segment are shown. For the *ψ* torsion of G25 and the *ϕ* torsion of S26, the distance between one of the two hydrogen atoms bonded to the α-carbon and a hydrogen atom in the lower monomer is shown (Note that the atom pair differs between the barrier top and the local minimum)

Types of torsion	Activation barrier (eV)	Atom pair	Interatomic distance at the barrier top (Å)	Interatomic distance at the local minimum (Å)
E22 (*ψ*)	0.49	H–H	1.7568	2.7152
		H–H	1.7432	1.9845
D23 (*ϕ*)	0.43	H–H	1.6629	5.9567
		H–H	1.9430	4.7694
D23 (*ψ*)	0.61	O–O	2.6464	3.1902
		O–O	2.8930	3.0023
V24 (*ϕ*)	0.43	O–O	2.2776	2.9519
		O–O	2.9646	2.9632
G25 (*ψ*)	0.05	H–H	2.8713	3.1348
S25 (*ϕ*)	0.50	H–H	2.3406	2.5905

To understand the origin of the activation barrier, we analyzed the structures at the activation barrier top and the local minimum on the PES. At the top of the activation barrier, we found that some interatomic distances between an atom contained in the sidechain near the torsional axis and an atom in the lower monomer become characteristically close, with many increasing at the local minimum. Table 1 lists these interatomic distances. For the *ψ* torsion of E22 and the *ϕ* torsion of D23, the hydrogen atoms of the E22 sidechain approach the hydrogen atoms in the lower monomer during the transition from the local minimum to the barrier top. For the *ψ* torsion of D23 and the *ϕ* torsion of D24, the oxygen atoms of the carboxyl group in the D23 sidechain approach the mainchain carbonyl oxygen atom of the 22–23 segment in the lower monomer.

In comparison with these torsions, for the *ψ* torsion of G25 and the *ϕ* torsion of S26, the sidechain near the torsional axis contains only a single hydrogen atom. Therefore, the intermolecular steric repulsion encountered during the twisted-to-flat conformational transition is greater in the *ψ* torsion of E22, *ϕ* torsion of D23, *ψ* torsion of D23, and *ϕ* torsion of V24 than in the *ψ* torsion of G25 and the *ϕ* torsion of S26. This steric hindrance accounts for the barrier height observed for the *ψ* torsion of E22, *ϕ* torsion of D23, *ψ* torsion of D23, and *ϕ* torsion of V24.

We next explain why the *ϕ* torsion of S26 still exhibits an activation barrier despite the little intermolecular steric repulsion. At the local minimum on the PES for the *ϕ* torsion of S26, an intramolecular HB forms between the mainchain carbonyl oxygen atom of the 25–26 segment and a hydrogen atom of the S26 sidechain. The stabilized twisted conformation due to this HB likely creates the barrier height, compensating for the energy increase in the latter part of the PES for the *ϕ* torsion of S26.

## 4 Discussion

We investigated the Aβ^20−34^ double-layer system and evaluated the PES associated with the interconversion between flat and twisted conformations. Based on the results, we discuss possible conformational changes in a realistic system. We first discuss the relevance of conformational interconversion studied here to molecular motion in an amyloid fibril, and then discuss the influence of the solution environment on the PES.

It is experimentally known that in the bulk region of the fibril, the Aβ adopts a cross-β structure and forms parallel in-register β-sheets with adjacent monomers, constituting a one-dimensional fibrillar morphology. Here, we consider a flat monomer located at the end of the fibril that is composed of parallel in-register β-sheets, and refer to it as a flat fibril end. The flat fibril end may be represented by the double-layer structure system studied here. Various fibril ends may be created by fibril fragmentation. Here, we assume two cases that happen to occur in fibril fragmentation: one in which the flat fibril end is created, and another in which a twisted conformation appears temporarily. In the following, we discuss conformational transitions in each case.

First, we discuss conformational transitions from the flat fibril end. In the present study, we considered twisted conformations arising from torsions around a single axis. Here, the β-sheet conformation was nearly preserved even in the region detached from the lower monomer. Our results showed that as the torsional motion involves greater disruption of intermolecular interactions (*e.g.*, HB and van der Waals forces) and intermolecular steric hindrance caused by sidechains, the activation barrier for the flat-to-twisted transition is higher. These results suggest that the detachment of a longer peptide chain while maintaining the flat conformation is unlikely to occur at the flat fibril end. Instead, we infer that the detachment of a shorter peptide chain involving torsions within the detached chain is more likely to occur.

Next, we discuss transitions from a twisted conformation. Our results showed that torsion occurring near glutamic acid and aspartic acid, which possess relatively bulky sidechains, resulted in relatively higher barriers for twisted-to-flat transition due to greater steric hindrance. In contrast, glycine gives a small sidechain (a single hydrogen atom). Note that the formation of additional intramolecular HB in a twisted conformation increases the barrier height. If such additional HB formation does not happen, torsion occurring near the glycine exhibits little steric hindrance and a low activation barrier. Our results agree with this tendency. A twisted conformation having a low activation barrier is expected to return to the flat conformation immediately. If one could experimentally determine which twisted conformation appears, transient twisted conformation given by the torsion occurring near the glycine, *ψ* torsion of G25, would be less likely to be observed.

The simulation in the present study does not consider the presence of the solvent around the Aβ^20−34^. Here, we discuss the possible effect of the solvent on the PES for the torsional motion of the Aβ^20−34^. The torsional transition from the flat conformation increases the number of sites in the Aβ^20−34^ where water molecules can form an HB, which can stabilize the twisted conformation. At the same time, it also increases the exposure of hydrophobic residues to solvents, which may be unfavorable for the realization of the twisted conformation due to the hydrophobic effect. We assume that the formation of HB between water molecules and the Aβ^20−34^ is a predominant effect in an aqueous solution environment. Then, in an aqueous solution, the stability of the twisted conformation is improved, thereby lowering the endothermic energy for the flat-to-twisted conformational transitions. If the presence of solvent molecules does not greatly disrupt the local conformation around the torsional axis, the activation barrier originating from the steric hindrance is expected to be maintained. We assume that the solvent effect is not strong enough to disrupt the local conformation around the torsional axis. Under such conditions, the intermolecular steric repulsion caused by the sidechain near the torsional axis is encountered during twisted-to-flat conformational transitions, allowing the twisted conformation to appear as local energy minima in the solution environment. A similar discussion has been presented on another molecular system, where two metastable states interconverting *via* torsional motion retain an activation barrier in the solution environment, although their relative stability changes due to the hydration effect.^38,39^

In actual fibril formation, the elongation process of the fibril proceeds *via* a consecutive addition of the monomer to the fibril end.^40^ Therefore, gaining deeper insight into the fibril formation mechanism requires understanding the detailed conformational characteristics of Aβ at the fibril end, as investigated in the previous study.^41^ Connecting our findings to understanding fibril-end conformation and ultimately, the fibril formation mechanism, is future work.

## 5 Conclusions

We theoretically investigated the structure and energetics of Aβ^20−34^ double layer system. Several locally stable structures of twisted conformations, in which a part of the peptide chain is directed upward due to torsion around a specific single bond, were identified by DFT-based structural optimization.

All flat-to-twisted conformational transitions were endothermic. In the flat conformation, closely formed HBs between monomers contribute to its stabilization. The transition to the twisted conformation partly breaks HBs, and the degree of destabilization (endothermic energy) is correlated with the number of broken HBs. In the *ϕ* torsion of D23, the repulsion of the carbonyl oxygen atoms between the upper and lower monomers destabilizes the twisted conformation. The van der Waals interactions, such as π-stacking and other intermolecular and intramolecular contacts of the hydrophobic sidechains, likely stabilize the flat conformation, and the loss of these interactions increases the endothermic energy required for the transition to the twisted conformation.

The PES connecting the flat and twisted conformation was computed using the NEB method. The activation barrier for twisted-to-flat conformational transitions was identified, representing the propensity of the former to revert to the latter. We compared the activation barriers for twisted-to-flat conformational transitions. Torsional motions involving the intermolecular steric hindrance caused by the sidechain near the torsional axis increase the activation barrier. If the additional formation of intramolecular HB occurs in a twisted conformation, an activation barrier for a twisted-to-flat transition is enhanced.

Our results suggest that, during the conformational transition from the flat conformation, detachment of a shorter chain involving torsions within the detached chain is more likely to occur than detachment of a longer chain maintaining the flat conformation.

If the additional HB formation in a twisted conformation does not happen, a twisted conformation resulting from torsions near small sidechains reverts to the flat conformation relatively easily. Linking the present results to the Aβ conformation at the fibril end and the mechanism of fibril formation remains a subject for future investigation.

## Conflicts of interest

No conflicts of interest to declare.

## Data Availability

The three-dimensional coordinate data of the conformational pathway obtained using the NEB calculation are available at the Open Science Framework at https://doi.org/10.17605/OSF.IO/WTYA5.
